# Influence of Ageing Time and Method on Beef Quality and Safety

**DOI:** 10.3390/foods12173250

**Published:** 2023-08-29

**Authors:** Sara Khazzar, Severino Segato, Giorgia Riuzzi, Lorenzo Serva, Elisabetta Garbin, Gabriele Gerardi, Sandro Tenti, Massimo Mirisola, Paolo Catellani

**Affiliations:** Department of Animal Medicine, Production and Health, University of Padova, 35020 Padova, Italy; sara.khazzar@studenti.unipd.it (S.K.); severino.segato@unipd.it (S.S.); giorgia.riuzzi@studenti.unipd.it (G.R.); elisabetta.garbin@unipd.it (E.G.); gabriele.gerardi@unipd.it (G.G.); sandro.tenti@unipd.it (S.T.); massimo.mirisola@unipd.it (M.M.); paolo.catellani@unipd.it (P.C.)

**Keywords:** beef, vacuum bag ageing, dry ageing, meat colour, meat tenderness, peroxide value, meat microbiological count

## Abstract

The effectiveness of dry ageing with regard to retaining meat quality is still subject to debate. At 4 d post mortem, samples of boneless strip loins were excised from young Charolais carcasses and then stored for a further 26 d in a cooler, either vacuum-packaged (VP) or dried-aged (DA). Loin samples were also dissected 7 d post mortem as a control treatment (CT). Chemical, instrumental and microbiological data (*n* = 18) were determined in *longissimus dorsi* and underwent ANOVA to estimate the differences in the ageing fixed factor split into two orthogonal contrasts: control vs. aged and VP vs. DA. Ageing loss (both surface dehydration and water purge) was greater in DA compared to VP samples, resulting in the lowest moisture content and highest crude protein and fat percentage in DA loins. The ageing method did not affect meat surface colour, except for redness, which had the lowest value in DA samples. Meat tenderness improved a similar amount following both VP and DA ageing treatments. Compared to the control, prolonged ageing raised both the peroxide value and the total microbial count, especially in DA samples, though both remained within the recommended limits. In summation, both ageing methods improved beef meat tenderisation, preserving its shelf life.

## 1. Introduction

Beef ageing is a combination of biochemical/biophysical processes that alter the muscle structural integrity and meat colour surface and palatability, which are naturally caused by a complex group of endogenous proteases and lipases [1,2]. Extending the ageing time has been reported to positively affect meat tenderness and juiciness [3]; moreover, the development of various compounds that enhance taste and aroma appears to be proportional to the duration of meat ageing [4]. However, the progression of ageing is still a highly debated topic, especially when comparing wet and dry ageing [5,6]. The use of muscle vacuum packaging with gas-impermeable plastic bags represents a feasible and attractive strategy to increase both quality and shelf-life without a subsequent deterioration of colour upon opening [7]. Though this is a costly alternative storage method, dry ageing under ambient conditions of critically controlled temperature, relative humidity and airflow is instead proposed to obtain premium tenderness and flavour primal/sub-primal cuts [2,8]. However, a prolonged dry ageing period without protective packaging materials can harden the muscle surface due to moisture evaporation, increasing trimming waste and the risk of growth of undesirable microorganisms such as bacteria, yeasts and moulds in non-edible crusts [9,10]. Moreover, beef dry ageing affects the extent of meat oxidation, which is related to exposure to light, metals, natural sensitisers and oxygen, and to the unsaturation degree of fatty acids [11,12]. To monitor total primary lipid peroxidation, the peroxide value has been suggested as a useful marker to evaluate the formation of compounds that are potentially harmful to human health [13]. Additionally, an extended storage time affects the microbial population on the surface of meat. In optimal anaerobic conditions such as vacuum-packaged muscle ageing, lactic acid bacteria (**LAB**) tend to be the prevalent taxon, exerting a protective competitive action toward the main spoilage and pathogenic microorganisms [14], while the prevalent microbial populations in dry-aged beef appear to be *Pseudomonas* spp. and *Enterobacteriaceae* [15]. However, the growth of these latter spoilage bacteria depends on the temperature and airflow setting, which influences the chemical characteristics of the drying crust [9,16]. 

The majority of studies testing the effects of a prolonged wet or dry ageing on selected meat quality traits have been carried out on highly marbled bone in primal cuts [6,15]. Meanwhile, very little published research has investigated how vacuum bag or dry ageing methods affect boneless lean primal cuts, such as those from French (e.g., Charolais and Limousine) or Italian (e.g., Romagnola) breeds reared in lowland-intensive systems [17,18]. In addition to the paucity of information regarding moderate and slight marbling beef muscles, the assessment of how ageing influences intrinsic qualities of beef, especially its tenderness and colour, remains imprecise. Indeed, there have been conflicting results in the literature, likely because the studies were based on different combined regimes (i.e., protective packaging material, refrigerated temperature, relative humidity and airflow) and storage lengths through either vacuum packaging or air ageing [10]. Therefore, the aim of this research was to explore the differences in beef *longissimus dorsi* quality and safety traits between two storage periods (carcass, 7 d vs. prolonged ageing, 30 d) and two ageing methods (vacuum packaged, **VP** vs. dry aged, **DA**). The idea behind this research was to investigate an alternative beef supply chain based on young bulls producing slight marbling muscles and on extending storage of boned-out primal cuts compared to the meat production from a conventional short-aged carcass.

## 2. Materials and Methods

### 2.1. Experimental Design, Carcasses and Muscle Sampling

At approximately 18 months of age, eighteen (*n* = 18) young Charolais males were slaughtered according to the current guidelines and in compliance with EC Regulation No. 1099/2009. The carcasses (384 ± 18 kg of cold weight) were refrigerated at 3 (±1) °C and conventionally aged for 4 days [19]. At the commercial slaughterhouse, before muscle samples were separated from both sides of each carcass, the pH was recorded in the longissimus muscle using the portable pH meter FG2-Five Go^TM^ equipped with a glass electrode suitable for muscle penetration and an automatic temperature compensator (Mettler-Toledo, Milano, Italy; calibration at pH 4.0 and 7.0), which was inserted into a small incision (approximately 4 cm depth) at the 4th–5th thoracic vertebra interface. After that (4 days post mortem), 36 boneless strip loin samples (muscle *longissimus dorsi*, muscle *spinalis dorsi*, muscle *trapezius* and muscle *semispinalis capitis*) were excised along the 5th–10th ribs both on the right (*n* = 18) and left (*n* = 18) side of the carcasses.

### 2.2. Ageing Treatments and Storage Weight Loss 

Boneless strip loin samples were randomly assigned to two ageing methods so that each carcass side was equally represented between the vacuum-packaged (VP) or dry-aged (DA) treatments. VP meat samples were vacuum sealed in medium-barrier bags—with a daily O_2_-transmission rate lower than 40 mL m^−2^ at 23 °C and 75% relative humidity [20]—using a CVV-41*n* vacuum packaging machine (Orved, Musile di Piave, Italy). Both DA and VP muscle samples were stored in a cooler at 3.0 ± 0.5 °C (average relative humidity, 75%; airflow, approximately 0.5 m s^−1^) until the 30th day post mortem. A short ageing process was also investigated and used as control treatment (**CT**); for this reference control treatment, joint samples of *longissimus dorsi* (3rd–4th ribs) were exceeded 7 days post mortem on both the carcass sides in order to collect *n* = 18 specimens (each composed by 2 left-side and 2 right-side subsamples of the loin) for VP and DA treatments. 

The initial weight loss (ageing loss due to surface dehydration and a water purge) of the VP and DA boneless strip loin samples was recorded by measuring the difference between their initial and post ageing weight and reporting it as a percentage ((initial weight − post ageing weight)/initial weight × 100) as suggested by Kim et al. [10]. The initial weight loss (e.g., storage weight loss) was determined without any sample trimming. Moreover, after removing from the vacuum bag, the VP aged boneless strip loin (VP treatment) was dry patted on paper towels and then reweighed.

### 2.3. Meat Quality Analyses

Trimmed *longissimus dorsi* samples of each treatment (*n* = 18) were used to perform meat chemical, instrumental and microbial analyses. As reported above, the treatments were based on the following ageing-methods–storage-periods (days post mortem): control (CT)—7 d on carcass, vacuum-packaged (VP)—30 d and dry-aged (DA)—30 d. 

#### 2.3.1. Drip Loss, pH and Colour

Meat drip loss was determined overnight using a gravimetric method, as suggested by Kim et al. [21], with a minor modification. Any visible fatty and/or connective tissues were trimmed off from a 2 cm-thick loin sample; next, the sample was weighted and placed into a polyurethane bag that was suspended with a hook in the grill shelf of a temperature control cooler. After 24 h of hanging at 2.5 (± 0.5) °C, the meat sample was blotted with a paper towel to remove any surface moisture and reweighted to determine the drip loss as a percentage. A portable pH meter (KnickPortamess 911, Berlin, Germany) equipped with a penetrating electrode (5 mm Ø conic tip, Crison 5232, Modena, Italy) was used to measure meat pH in triplicate. Water activity (a_w_) was determined using an AquaLab CX2-Decagon apparatus (Decagon Devices, Pullman, WA, USA). 

A further 2 cm-thick loin sample was air bloomed at 3 ± 1 °C for approximately 60 min, and then the meat surface CIE L*a*b* (L*, lightness; a*, redness; and b*, yellowness) colour coordinates were recorded in five replicates using a Konica Minolta CD-600 visible spectrophotometer (Konica Minolta, Osaka, Japan) [22], with D65 as a light source and standard observer of 10° [23]. The a* and b* coordinates were used to calculate C* (chroma or vividness of H*) and H* (hue angle or the degree to which a colour stimulus can be described) using the formulas [24] given in Equations (1) and (2):

(1) $$C^{*}=\sqrt{{\left(a^{*}\right)}^{2}+{\left(b^{*}\right)}^{2}}$$

(2) $$H^{*}=arctangent\left(\frac{b^{*}}{a^{*}}\;\right)\times \left(\frac{360{}^{\circ}}{2\times \pi }\right)$$

#### 2.3.2. Cooking Loss and Instrumental Tenderness 

To determine cooking losses, a 2.5 cm-thick steak was wrapped individually in a polyurethane bag, heated in a thermostatic water bath (50 min, 72 °C) and then cooled under running tap water for at least 40 min. After cooling, 8 cylindrical subsamples were excised from the cooked steak, parallel to the meat fibres by means of a steel borer (Ø 1.25 cm), to determine the instrumental tenderness with a dynamometer TA-HDi texture analyser (Stable Micro Systems, Surrey, UK) equipped with a V-slot Warner–Bratzler blade (load rate of 1 mm/s). The meat toughness was expressed as the maximum shear force (**WBSF**) applied (Newton, N) with the instrument to cut the meat sample. 

#### 2.3.3. Proximate Composition and Peroxide Value

Each residual trimmed loin subsample was split into aliquots, which were submitted to the following chemical analyses based on AOAC procedures [25]: (i) moisture was analysed overnight at 103 °C (# 950.46); (ii) crude protein (N × 6.25) according to the Kjeldhal procedure (# 981.10); (iii) fat content via the Soxhlet procedure with petroleum ether as a solvent (# 991.36); and (iv) ash (mineral) in a muffle furnace by heating the samples at 550 °C for 4 h (# 920.153). The peroxide value was determined based on fat samples (approximately 1 g) that were solubilised with a mixture of acetic acid/chloroform (3/2). After the addition of 0.5 mL of a saturated solution of KI and incubation in the dark for 2 min, the elemental iodine developed by the hydroperoxides was titrated by using a solution of 0.01 M sodium thiosulphate in the presence of starch as an indicator [26]; the results were expressed as µg of active O_2_ g^−1^ fat.

### 2.4. Microbial Analysis

For the microbiological analyses, 20 g of meat was aseptically sampled from each loin and placed into a sterile stomacher bag containing 180 mL of buffered peptone water (BPW), and then homogenised using a stomacher (Stomacher 400 circulator, Seward, Worthing, UK) for 2 min. Serial dilutions were performed by adding 1 mL of the diluted sample to 9 mL of BPW (decimal logarithmic scale dilutions) followed by incubation in specialised bacterial growth agar media according to the times and temperature stated for the following methods: total viable count (**TVC**; ISO 4833:2003), *Pseudomonas* spp. (ISO 13720:2010), *Enterobacteriaceae* (ISO 21528:2017) and lactic acid bacteria (ISO 15214:1998). Microbial counts were reported as log colony-forming units (log_10_ CFU g^−1^ meat). 

### 2.5. Statistical Analysis

All the statistical analyses were computed using SAS 9.4 software (SAS Institute Inc., Cary, NC, USA). For the whole dataset, the assumption of normality and variance homogeneity was assessed using the Shapiro–Wilk test (PROC UNIVARIATE) and considering a threshold of 0.90 as a limit for a normal distribution. Meat chemical, instrumental and microbiological data were submitted to ANOVA (PROC-GLM), adopting a linear model that considered the fixed factor of ageing split into two orthogonal contrasts: CT vs. (VP + DA)/2 and VP vs. DA. The hypothesis of the linear model on normal distribution of the residuals was graphically assessed. The significance of the tests was set at *p* < 0.05.

## 3. Results

### 3.1. Ageing and Drip Losses, Proximate Composition, Colour and Texture

On the 4th day post mortem, before the strip loin sampling, the carcass loin pH was determined and the average value was equal to 5.63 ± 0.07, suggesting a normal muscle acidification range [18,27]. Moreover, none of the carcasses showed a DFD potential risk condition (pH ≥ 5.80). After 30 d of ageing, boneless DA muscle beef samples had greater initial weight loss (e.g., ageing loss) compared to the VP ones (this outcome is not statistically supported since it was not measured in CT). There were no significant differences in meat drip loss across the treatments (Table 1). The meat (m. *longissimus dorsi*) proximate composition is reported in Table 1. As expected, the DA samples had the lowest moisture content and, as a consequence, the air ageing led to a higher concentration of crude protein and lipids than the vacuum packaged ageing (orthogonal contrast VP vs. DA, *p* < 0.05). 

The instrumental colour coordinates are reported in Table 2. Compared to the control (CT), the prolonged ageing induced a significant decrease in lightness (L*) and redness (a*), while the yellowness (b*) was not affected (Table 2). In the DA treatment, the lowest value of a* was the main difference detected in surface meat colour compared to VP (VP vs. DA, *p* < 0.05). The chroma (C*) did not change across treatments, while hue angle (H*) was higher (larger angle) in both VP and DA meat samples compared to the CT. The prolonged ageing period significantly reduced the cooking loss in VP and DA (Table 2); in fact, the highest loss percentage was observed in CT samples (orthogonal contrast CT vs. (VP + DA/2), *p* < 0.05)). Similarly, the texture measured via the Warner–Bratzler test was significantly lower for both the ageing methods in comparison with the CT treatment (Table 2). 

### 3.2. Chemical Traits and Microbial Status 

The storage period significantly influenced the beef loin pH (orthogonal contrast CT vs. (VP + DA/2), *p* < 0.05)), which, in contrast, was not altered by the ageing method (Table 3). Regarding the water activity, no differences were detected across the treatments (Table 3). The DA treatment had the highest peroxide value, as displayed with the significance (*p* < 0.01) of the orthogonal contrast VP vs. DA, while the short carcass ageing (CT treatment) had the lowest peroxide value (Table 3). 

The number of bacteria (total viable count, TVC) significantly (orthogonal contrast CT vs. (VP + DA/2), *p* < 0.01)) increased during the storage period, while no notable differences were detected between the two ageing methods. However, the microbial community was quite diverse across the experimental treatments with a prevalence of LAB in VP samples and of *Pseudomonas* spp. in the DA ones (Table 3).

## 4. Discussion

The experimental design was selected to assess the influence of both the storage period and ageing methods on beef loin chemical, instrumental and microbial traits. The main goal was a comparison among 7-day carcass aged samples (CT—control treatment) and 30-day aged samples either vacuum packaged (VP) or dry aged (DA) in an environmental (T, humidity and airflow) control cooler. Moreover, the trial aimed to evaluate the effects of a 1-month ageing process on smaller primal cuts (bone-out), which take up less space in the cooler and allow for easier handling. To assist ageing processors interested in exploring the potential benefits of an ageing method applied to smaller and lighter (sub)primal cuts, this study assessed the possible differences in meat quality and safety between two extended ageing methods versus a conventional short carcass ripening.

The initial weight loss (e.g., surface dehydration and a water purge) and meat drip loss, together with the consequent change in concentration of chemical constituents, are in agreement with several similar trial studies [2,28,29]. As expected, lower initial weight loss was observed in the VP strip loins compared to the DA ones [10]. The observed ageing loss was higher than that reported in a similar study performed by Di Paolo et al. [18] on a primal retail cut (bone-in *longissimus dorsi*, ribeye steak) from Charolais. The higher weight loss recorded in our study was likely due to both the high T setting in the cooler (3° vs. 1° C), which led to a greater amount of water evaporation, and the absence of bone that could limit the surface dehydration [30]. During dry ageing, water is transferred via diffusion from the interior to the surface of the muscle, where most of it evaporates. Therefore, as muscle tissues lose water, the chemical constituents become more concentrated, often resulting in higher protein and lipid percentages. However, despite avoiding the water evaporation, the negative pressure and the physical squeezing due to the vacuum packaging shrinkage seemed to increase the ageing weight loss [31]. Indeed, the intramuscular fat content recorded in this trial remained very low, although it adhered to the standards of this French breed reared in extensive systems in the north of Italy [32,33].

Although the a* (indication of redness) and b* (indication of yellowness) coordinates reflect relevant colour changes, a comprehensive explanation of the effects of ageing on meat surface colour should be based on a three-dimensional space considering lightness, hue and saturation properties [20]. The main colour difference detected between CT and the more aged samples appeared to indicate a reduced blooming ability after the prolonged storage period, likely due to the higher pH and lower moisture content. The DA treatment had the lowest value of redness (a*), while both lightness (L*) and chroma (C*) remained unchanged. As a statistical tendency (orthogonal contrast VP vs. DA, *p* = 0.064), the VP loins showed a lighter colour—a greater lightness, L*, value—compared to the DA counterpart. This is a meaningful outcome from a biochemical point of view, as it might be associated with greater light reflection due to a greater moisture content. This surface colour difference, nonetheless, has a limited impact on the meat’s visual acceptability, even for trained beef quality experts [34]. Chroma represents the colour intensity, describing its vividness or dullness and, therefore, it is an effective indicator of the oxygenation of meat recently exposed to the air [35]. The fact that C* did not change across the treatments indicates that the inner meat layers in the DA samples were not characterised by a higher myoglobin auto-oxidation, and when exposed to the air, they displayed a similar vivid colour compared to the VP meat. The higher H* values recorded for the VP and DA samples suggest that prolonged storage altered the meat colour, since a high level of the hue angle (H*) seemed to be associated with a lower red perception for consumers. 

Cooking loss was higher in the CT loins compared to their aged counterparts, an outcome that probably resulted from their higher moisture content. However, DA loins showed a greater total water loss, calculated by summing initial, drip and cooking losses together, confirming that a dry ageing process usually results in a lower yield compared to sealed vacuum bag (wet ageing) muscle ripening [5,10]. As expected [19,28], both the ageing methods significantly reduced the cutting shear force, increasing meat tenderness; this was likely due to the degradation of specific myofibrillar protein by calpain proteases, a post mortem proteolysis process that occurs during extended storage [36,37]. As reported in the literature [10,16,38], no significant impact of dry ageing on beef loin tenderness was observed compared to the VP counterpart. Nevertheless, this experimental finding could derive from the adopted processing regimes, since substantially different meat quality attributes could be obtained from different temperature, humidity air and/or air-velocity cooler settings [10].

As found by Bogdanowicz et al. [19], the loin pH increased during the ageing period by approximately 0.10 units compared to the value recorded in CT samples. This experimental finding was probably due to the activation of microflora and endogenous enzymes accompanied by protein degradation, which resulted in the release of ammonia and sulphur compounds; this led to a significant increase in the meat pH value [39]. The average lipid peroxidation estimated as the peroxide value was similar to that reported by Boselli et al. [13] in a trial on the effects of the type of packaging and different mass to surface ratio on beef meat lipid oxidation. Nevertheless, it was lower than the amount observed in small pieces of femur beef packed in polyethylene bags and stored for 28 d at 4 °C [40]. Peroxides are the primary product created during lipid peroxidation and might be further broken down into low-molecular-weight products, including many favourable aroma compounds (e.g., ketones, aldehydes, esters, etc.). However, the oxidation of pigments and lipids could impair the shelf life of fresh meat because they could cause surface discolouration and rancid flavours [12,41]. Although the subcutaneous fat surface of the beef loins was trimmed and discarded, the total microbial community was affected by both the storage period and the ageing method, as VP and DA had higher *Enterobacteriaceae* and LAB amounts compared to their CT counterparts. It is well known that *Pseudomonas* spp. is inhibited under anoxic conditions and is CO_2_ sensitive [42]. Therefore, regarding the comparison between ageing methods and due to this selective action, spoilage bacteria under the aerobic conditions occurring in DA samples are dominated by *Pseudomonas* spp. Meanwhile, the facultative anaerobic LAB are the dominating spoiling population detectable after VP storage [2]. The observed prevalence of *Pseudomonads* spp. and *Enterobacteriaceae* is consistent with the literature, in which *Pseudomonas* spp. was identified as a core taxon in dry-aged meat, especially in bone-in samples [43]. Complying with the EC legislation (Regulation EC No. 2073/2005), the average TVC was below 5 log CFU/g across the treatments, suggesting the use of acceptable processing procedures with a low storage temperature set up in this experiment.

The chemical and microbiological data from this trial suggested that meat of acceptable quality and sanitary traits can be obtained through prolonged ageing, as it has no negative impact on primary lipid peroxidation and microbial spoilage. The range of temperature, humidity and light conditions in the cooler, as well as the mechanical and barrier properties of the films for vacuum packaging, are controlling key factors affecting meat safety and shelf life during both ageing and retail display [13,44,45]. However, beyond the scope of this research, further investigation is necessary to develop an in-depth understanding of the safety and sustainability of extended ageing in the fresh beef meat industry.

## 5. Conclusions

Compared to a short carcass storage period, the extended ageing methods affected meat quality, making it more tender and richer in crude protein and lipid content, while the meat surface lightness and colour stability was only moderately affected under the dry-ageing chilled conditions. From a commercial point of view, the DA method seemed to be the least effective, since it resulted in the lowest meat yield due to the highest moisture loss, especially during ageing. Limited, yet significant, increases in the peroxide value and microflora in the extendedly aged samples were observed. However, both vacuum packaging and air ageing did not appear to alter the extendedly chilled meat shelf life. Therefore, when considering a longer storage period, both the investigated ageing methods allowed us to enhance meat tenderness and retain its fresh attractive appearance, although further studies should consider a more holistic approach that includes other meat safety and sensory perceived variables.

## Figures and Tables

**Table 1:** Initial weight loss, meat (*longissimus dorsi*) drip loss and proximate composition (on wet weight basis) according to the storage period (days post mortem) and ageing method ^1^.

Item	Control	Ageing Method		SEM	Orthogonal Contrasts	
	(*n* = 18)	VP (*n* = 18)	DA (*n* = 18)		CT vs. (VP + DA)/2	VP vs. DA
Initial weight loss (%) ^2^	n.e.	3.4	8.1	-	-	-
Drip loss (%)	1.9	2.2	2.4	0.2	0.123	0.184
Moisture (%)	74.5	72.7	71.1	0.5	0.001	0.028
Crude protein (%)	21.6	22.8	23.9	0.4	0.001	0.025
Fat (%)	2.8	3.2	3.6	0.2	0.001	0.021
Ash (%)	1.1	1.3	1.4	0.1	0.001	0.238

^1^ Control treatment (CT), 7 d on carcass; vacuum packaged (VP) and dry aged (DA), 30 d (4 carcass and 26 cooler). ^2^ Initial weight loss (ageing loss due to surface dehydration and water purge) was not estimated (n.e.) in control treatment.

**Table 2:** Instrumental meat (*longissimus dorsi*) quality traits according to the storage period (days post mortem) and ageing method ^1^.

Item	Control	Ageing Method		SEM	Orthogonal Contrasts	
	(*n* = 18)	VP (*n* = 18)	DA (*n* = 18)		CT vs. (VP + DA)/2	VP vs. DA
L* (lightness)	37.8	36.3	35.5	0.4	0.035	0.062
a* (redness)	18.9	18.4	17.3	0.4	0.032	0.012
b* (yellowness)	9.7	10.2	9.8	0.3	0.823	0.188
C* (chroma)	21.2	21.1	19.8	0.3	0.423	0.157
H* (hue angle)	27.1	29.1	29.6	0.6	0.038	0.583
Cooking loss (%)	32.1	29.2	28.9	0.6	0.038	0.704
WB shear force (N)	50.2	39.1	42.3	1.9	0.021	0.406

^1^ Control treatment (CT), 7 d on carcass; vacuum packaged (VP) and dry aged (DA), 30 d (4 carcass and 26 cooler).

**Table 3:** Meat (*longissimus dorsi*) chemical traits and microbial count according to the storage period (days post mortem) and ageing method ^1^.

Item	Control	Ageing Method		SEM	Orthogonal Contrasts	
	(*n* = 18)	VP (*n* = 18)	DA (*n* = 18)		CT vs. (VP + DA)/2	VP vs. DA
pH	5.54	5.65	5.60	0.03	0.032	0.224
Water activity (a_w_)	0.99	0.99	0.98	0.002	0.548	0.183
Peroxide value (μg O_2_/g fat)	10.5	12.6	18.4	1.3	0.009	0.001
Microbial count (log_10_ CFU/g)						
Total viable count	2.5	3.8	4.5	0.5	0.001	0.242
*Enterobacteriaceae*	1.0	1.7	1.9	0.3	0.056	0.218
*Pseudomonas* spp.	0.9	0.6	1.8	0.2	0.423	0.001
Lactic acid bacteria	0.2	1.0	0.4	0.3	0.045	0.001

^1^ Control treatment (CT), 7 d on carcass; vacuum packaged (VP) and dry aged (DA), 30 d (4 carcass and 26 cooler).

## Data Availability

None of the data were deposited in an official repository.
